# Why culture remains the gold standard: about two cases of *Staphylococcus aureus* bloodstream infections

**DOI:** 10.1128/spectrum.00636-26

**Published:** 2026-07-24

**Authors:** Sylvain Meyer, Thomas Lafon, Olivier Barraud

**Affiliations:** 1 Université de Limoges, Inserm, CHU Limoges, RESINFIT, U1092, Limoges, France; 2 Service d’Accueil des Urgences, CHU Limoges, Limoges, France; Second Affiliated Hospital of Soochow University, Suzhou, China

**Keywords:** bloodstream infection, genotype, phenotype, whole genome sequencing, *Staphylococcus aureus*

## LETTER

Genomics is now widely used as a first-line routine test to predict bacterial identification and/or antimicrobial resistance. Genomics has become the reference for many bacteriological analyses, overpassing conventional microbiology and phenotypic tests because of its time-saving advantages and greater sensitivity. Nevertheless, PCR can, in rare cases, fail to amplify the targeted gene, especially if modifications in the target sequence, such as mutations, insertions, or deletions are present. Here, we present two clinical cases of *Staphylococcus aureus* bloodstream infections where amplification of the protein A encoding gene (*spa*) failed to identify *S. aureus* and amplification of the methicillin resistance gene (*mecA*) failed to detect methicillin susceptibility.

**Fig 1:**
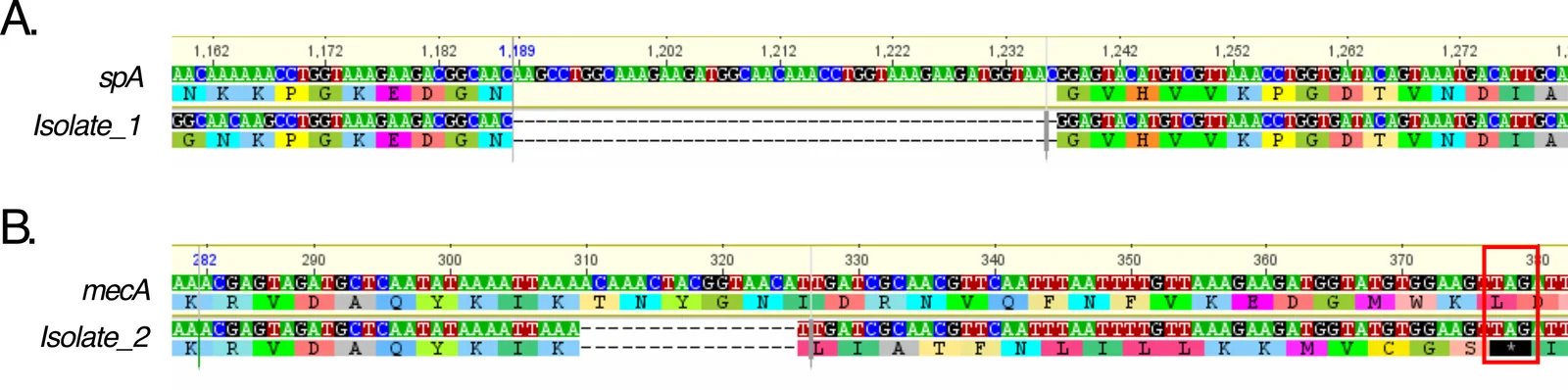
Comparison of *spa* gene (**A**) and *mecA* gene (**B**) of sequenced *S. aureus* isolates against reference sequence NZ_CP027476.1 using Geneious Prime software. The stop codon is highlighted in red.

The first patient was admitted to the emergency unit of Limoges University Hospital in August 2023 with septic shock. Blood culture became positive in 8 hours with gram-positive cocci. PCR using the XpertMRSA/SA Blood Culture Kit (Cepheid) was performed and showed absence of amplification for the *spa* and *mecA* genes. Identification performed from culture using the MALDI Biotyper System (Brucker) confirmed the presence of *S. aureus* (LIM207). In front of this discrepancy, whole genome sequencing of LIM207 was performed using the Ion GeneStudio platform (Thermo Fisher Scientific), as previously described (1). This ST121 isolate was found to have a deletion of 48 nucleic acids in the *spa* gene (Fig. 1A). The exact region amplified by the PCR kit not being known, it is expected that the primers or probe of this qPCR target this deleted region. The patient died 1 day after admission despite receiving adapted antibiotic therapy from the time of admission.

The second patient was admitted to the same emergency unit with a suspicion of dermohypodermitis without septic shock. Blood cultures became positive in 9 hours with gram-positive cocci. The same XpertMRSA/SA Blood Culture Kit showed both *spa* and *mecA* amplifications in favor of methicillin-resistant *S. aureus*. Bacterial culture confirmed the *S. aureus* identification, but antimicrobial susceptibility testing (AST) showed methicillin-susceptible *S. aureus* (MSSA). Confirmation of methicillin susceptibility was performed using three different tests: cefoxitin disk diameter >22 mm, oxacillin MIC ≤0.25 mg/L, negative cefoxitin test on the Vitek2 AST-P631 card (bioMérieux), and negative PLP2a immunochromatography using the ClearviewPBP2a SA Culture Colony Test (Abbott). WGS showed this ST5 *S. aureus* (LIM208) had an SCCmec type IV with a deletion of 16 nucleic acids in the *mecA* gene at position 309, leading to an early stop codon of this PLP2A protein at position 121 (Fig. 1B). Because of this discrepancy, the patient was first treated with linezolid and gentamicin, with a switch to cefazolin after MSSA confirmation. The patient died a couple of weeks later due to a massive *S. aureus* endocarditis.

In conclusion, these two cases highlight the limit of PCR and the importance of systematic conventional culture and AST to confirm diagnosis. Deletion of part of the genome has been previously reported in *S. aureus* (2), but small deletions inside the *spa* or *mecA* genes are very uncommon, with a potential impact on PCR results or gene expression (3). These two *S*. *aureus* isolates are atypical and have never been described before. We are therefore alert to the eventuality of false positive/negative PCR results targeting these genes with similar isolates. Use of WGS in clinical practice can help to solve such cases, even though time-to-result and costs should be considered.

## Data Availability

Both genomes were deposited and are available under accession number PRJNA1412556 on the GenBank database.

## References

[1] Meyer S, Hernandez-Padilla AC, Fedou A-L, Daix T, Chainier D, Ploy M-C, Vignon P, François B, Barraud O. 2024. Longitudinal two-year comparative genomic analysis of respiratory *Staphylococcus aureus* isolates from intensive care unit mechanically ventilated patients. J Hosp Infect 154:37–44. doi:10.1016/j.jhin.2024.09.004 39278267

[2] Baum C, Haslinger-Löffler B, Westh H, Boye K, Peters G, Neumann C, Kahl BC. 2009. Non-*spa*-typeable clinical *Staphylococcus aureus* strains are naturally occurring protein A mutants. J Clin Microbiol 47:3624–3629. doi:10.1128/JCM.00941-09 19759222 PMC2772612

[3] Lee G-H, Pang S, Coombs GW. 2018. Misidentification of *Staphylococcus aureus* by the cepheid Xpert MRSA/SA BC assay due to deletions in the *spa* gene. J Clin Microbiol 56:e00530-18. doi:10.1128/JCM.00530-18 29743311 PMC6018334

